# Membranous Dysmenorrhea: A Case Series

**DOI:** 10.1100/tsw.2007.277

**Published:** 2007-11-26

**Authors:** Hatim A. Omar, Shawn J. Smith

**Affiliations:** Division of Adolescent Medicine, Department of Pediatrics, University of Kentucky, Lexington, KY 40536, USA

**Keywords:** dysmenorrhea, menstrual disorders, contraception, vaginal bleeding, endometrium

## Abstract

The purpose was to illustrate the variability of hormonal contraception of patients that presented with membranous dysmenorrheal. A case analysis chart review was completed on six patients referred to a Pediatric Gynecologist in an academic setting. In each case the patient underwent a thorough pelvic and bimanual exam. Following the initial presentation, each patient continued to be followed on a regular visits. Cases: Two were using the transdermal contraceptive patch and oral contraceptive, but following the expulsion of decidual cast, they were both placed on depot medroxyprogesterone acetate (DMPA) without further complications. Three of the six cases were on DMPA prior to the similar occurrence of membranous dysmenorrheal and following this incident, continued on DMPA without further problems. The final case was on the transdermal patch prior to decidual cast expulsion and remained on this form of hormonal contraception without further complications. These cases indicate that membranous dysmenorrheal is not limited to the use of DMPA.

### INTRODUCTION

Membranous dysmenorrhea is a clinical entity originally reported in the literature decades ago. It is a term that involves sloughing of the endometrium either in one piece retaining the shape of the uterus, or in several membranous pieces[1]. Further reports of this still somewhat poorly described disorder have been published to hypothesize various etiologies, specifically identifying depot medroxyprogesterone (DMPA) as a causative agent.

We are reporting six cases with various presentations. Unique to this case study is not only the form of hormonal contraception of the patient prior to experiencing membranous dysmenorrheal, but also the choice of contraception following the event.

In each case the patient was referred to a pediatric gynecologist at an academic setting for a thorough pelvic and bimanual exam. In each case there was a normal size uterus and no evidence of a mass. Following the incident, the patient was closely monitored with regular follow up clinic visits.

### CASE REPORTS

#### Case 1

A 12-year-old with cerebral palsy, epilepsy, and mental retardation was placed on norelgestromin/ethinylestradiol transdermal patch (Ortho evra) for management of dysmenorrhea. Following a night of intractable seizures, she began experiencing vaginal bleeding and was evaluated by her primary care physician. During the clinic visit, she experienced colicky abdominal pain and subsequently vaginally expelled tissue. The tissue was intact and grossly retained the triangular shape of the uterus (Figs. 1, 2). The histologic examination showed decidual tissue. Following this event, Depot Medroxyprogesterone Acetate (DMPA) replaced the transdermal patch, which has successfully treated her dysmenorrhea. She has had no further events or complications during a 3 years follow-up period.

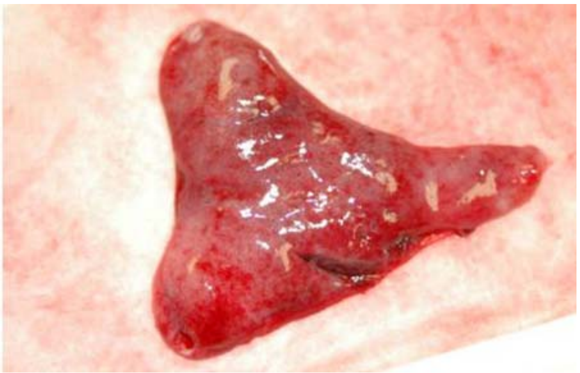

**Figure 1.** The uterus shaped tissue discussed in Case 1

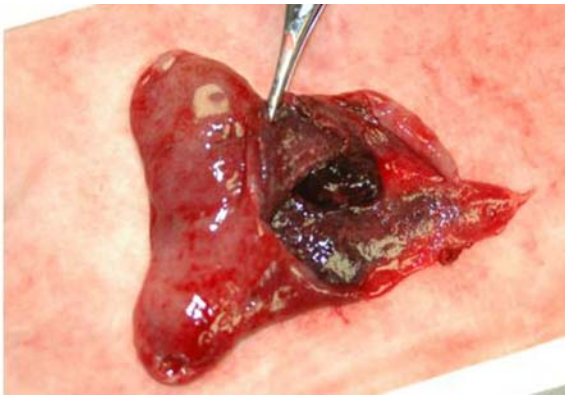

**Figure 2.** The tissue from Case 1, open and showing blood clots and endometrium

#### Case 2

A 13-year-old was placed on DMPA for dysmenorrhea. After one cycle of treatment she had severe, colicky abdominal pain and experienced vaginal expulsion of intact tissue (Fig. 3). Despite this event she was continued with DMPA and is currently in her fourth year of treatment. Since this event she has had no subsequent episodes and her dysmenorrhea is well controlled.

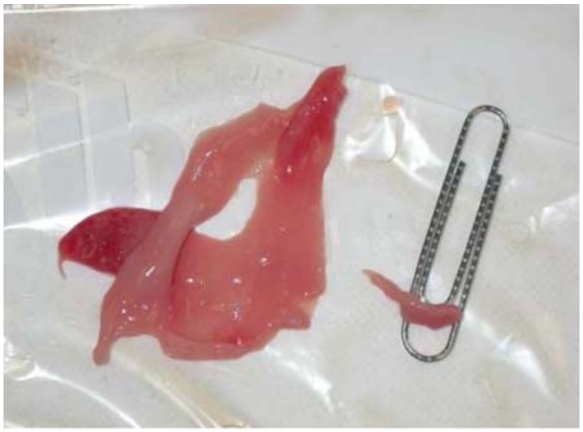

**Figure 3.** The tissue from Case 2, partial endometrium

### Case 3

A 15-year-old was placed on the transdermal patch for contraception. During the second week of the fifth cycle she experienced severe colicky abdominal pain and subsequent vaginal expulsion of pieces of tissue. Similar to previous cases she continued the contraceptive transdermal patch without further without further complications and her dysmenorrhea has been well treated.

### Case 4

A 16-year-old was placed on DMPA for contraception. During the fifteenth month of use she had severe abdominal pain and vaginal bleeding lasting for three days. On the third day she vaginally expelled pieces of tissue. She was continued on DMPA and is currently in her third year of usage and has had no subsequent events or dysmenorrhea.

### Case 5

A 17-year-old was placed on Ortho Tri-Cyclen (norgestimate 0.18 mg, 0.215 mg, 0.25 mg/ethinyl estradiol 35 mcg). During her seventh month of usage, she reportedly missed two pills and experienced withdrawal bleeding for two days. On the second day she had severe colicky abdominal pain and vaginal tissue expulsion. The oral contraceptive was discontinued on and subsequently She was started on DMPA. She is currently in her second year with DMPA without complications.

### Case 6

A 16-year-old gravida 1, para 1 began DMPA at her six-week postpartum visit. Two months after her initial DMPA injection she had abdominal pain and vaginally expelled pieces of tissue. She was given a DMPA injection during this same visit and is now currently at the end of her second year without complications. Histologic examination of the expulsed material in all cases was consistent with decidual tissue.

## DISCUSSION

Membranous dysmenorrhea is an entity that has been reported periodically in the last few decades through case reports and studies. Unfortunately, the etiology remains unknown. The theory of hyperprogesteronism has been hypothesized and cases have been observed of decidual cast expulsion following progesterone[2]. Other theories proposed by Greenblatt et al include an overall increase in the secretion of progesterone and estrogen, subsequent thickening of the endometrium and resultant incomplete desegregation with expulsion of tissue; excessive development of the spiral arteries, with subsequent vasodilatation followed by vasoconstriction and then shedding of this overdeveloped endometrium; and finally, they have proposed that this poorly defined entity may represent an accentuation of normal menses. A case report was published describing imaging correlating with membranous dysmenorrhea[3]. The possibility of identifying this disorder may prove beneficial in the future. Recently there has been a suggestion that membranous dysmenorrhea is correlated with DMPA[4]. Case 1 and Case 5 were on the transdermal patch and Ortho Tri-Cyclen respectively, and after experiencing severe, colicky abdominal pain and subsequent expulsion of tissue, both were then placed on DMPA without further problems. Case 2, 4 and 6 were on DMPA prior to having experienced a similar occurrence of pain followed by vaginal tissue expulsion. However, each was continued on her regular schedule of DMPA without further incidences. Similarly, Case 3 was also successfully continued on her method of contraception, the transdermal patch, despite having experienced severe pain associated with vaginal tissue expulsion.

These cases clearly contradict the cause/effect between membranous dysmenorrhea and DMPA due to the diversity of circumstances presented by our patients. It is unclear what cause this unusual type of dysmenorrhea and more studies are needed to clarify the etiology and pathophysiology of this disease. This study also shows that there are usually no negative long term consequences to this illness and that in most cases, the patient can continue to use the contraceptive method without problems.
